# THE SIGNIFICANCE OF IDENTIFYING A TIPPING POINT

**DOI:** 10.1093/geroni/igz038.2222

**Published:** 2019-11-08

**Authors:** Lorraine Martin-Plank, Lori Martin-Plank, Beverly Heasley, Cheryl L Lacasse, Linda R Phillips, Jian Liu

**Affiliations:** 1 University of Arizona, Tucson, Arizona, United States; 2 University of Arizona, College of Nursing, Pipersville, Pennsylvania, United States; 3 Arizona Center on Aging, Tucson, Arizona, United States; 4 University of Arizona, College of Nursing, Tucson, Arizona, United States; 5 University of Arizona, Center on Aging, Tucson, Arizona, United States; 6 University of Arizona, Department of Systems and Industrial Engineering, Tucson, Arizona, United States

## Abstract

Defining a Tipping Point as seemingly abrupt, severe, and absolute vividly illustrates the end result of the consequence of not responding to impending events. Failure to detect an impending tipping point can result in unplanned consequences including falls, fractures, emergency care, a change in family caregiving situation, and a cascade of events leading to devastating permanent changes in chosen living arrangements or even death to the aging adult and the family caregiver. Proactive monitoring with a wearable device that can detect subtle changes in daily routines is a valuable tool, which caregiving families can use for decision support in accessing long term support services or planning for change in environment or level of care. Using this definition of tipping points and describing early changes can also be a valuable tool for clinicians when communicating potentially dangerous situations to the older adult and caregivers in the home or senior residential setting.

